# Societal and individual burden of illness among fibromyalgia patients in France: Association between disease severity and OMERACT core domains

**DOI:** 10.1186/1471-2474-13-22

**Published:** 2012-02-17

**Authors:** Serge Perrot, Caroline Schaefer, Tyler Knight, Meghan Hufstader, Arthi B Chandran, Gergana Zlateva

**Affiliations:** 1 Service de Médecine Interne et Thérapeutique, Consultation de la Douleur, Hôtel Dieu Hospital, Université Paris Descartes, Paris, France; 2 Covance Market Access Services Inc., Gaithersburg, MD, USA; 3 Pfizer Inc, New York, NY, USA

**Keywords:** Burden of illness, Fibromyalgia, France, Quality of life, Healthcare costs, Outcome Measures in Rheumatology Clinical Trials (OMERACT)

## Abstract

**Background:**

Patients with fibromyalgia (FM) report widespread pain, fatigue, and other functional limitations. This study aimed to provide an assessment of the burden of illness associated with FM in France and its association with disease severity and core domains as defined by Outcome Measures in Rheumatology Clinical Trials (OMERACT) for FM.

**Methods:**

This cross-sectional, observational study recruited patients with a prior diagnosis of FM from 18 community-based physician offices in France. Patients completed questions about FM impact (Fibromyalgia-Impact Questionnaire [FIQ]), core symptoms (defined by OMERACT), health-related quality of life (EQ-5D), current overall health status (rated on a scale from 0 to 100), productivity, treatment satisfaction, and out-of-pocket expenses related to FM. Site staff recorded patients' treatment and health resource use based on medical record review. Costs were extrapolated from 4-week patient-reported data and 3-month clinical case report form data and calculated in 2008 Euros using a societal perspective. Tests of significance used the Kruskal-Wallis test or Fisher's Exact test where *P *< 0.05 was considered significant.

**Results:**

Eighty-eight patients (mean 55.2 y; female:male 74:14) were recruited. The majority of patients (84.1%) were prescribed medications for FM. Patients mainly described medications as a little/not at all effective (40.0%) or somewhat effective (52.9%). Current Overall Health rating was 52.9 (± 17.8) and FIQ total score was 54.8 (± 17.3). FIQ total score was used to define FM severity, and 17 patients scored 0- < 39 (mild FM), 33 patients 39- < 59 (moderate FM), and 38 scored 59-100 (severe FM). As FM severity level worsened, patients had poorer overall health status and perceived their prescription medications to be less effective. Average cost/FM patient was higher for severe (€10,087) vs. moderate (€6,633) or mild FM (€5,473); however, the difference was not significant.

**Conclusions:**

In a sample of 88 patients with FM from France, we found that FM poses a substantial economic and human burden on patients and society. FM severity level was significantly associated with patients' health status and core symptom domains.

## Background

Fibromyalgia (FM) is a chronic disorder characterized by persistent, widespread pain [1]. FM patients may also report fatigue, sleep disturbance, anxiety, and depression [2-4]. Although estimates vary, FM has been reported to affect up to 6% of the general European population [5-7]. However, one community study in France estimated the point prevalence of FM to be lower at 1.4%, which translates to ~680,000 patients [8].

Confirming a diagnosis of FM is often difficult because there is no specific diagnostic test to clearly validate the disorder. Indeed, FM is often diagnosed by ruling out other conditions, such as rheumatoid arthritis, lupus, and multiple sclerosis [9]. Various professional bodies have issued guidelines to assist diagnosis of FM such as screening tools [10] or diagnostic criteria, such as those recently issued by the American College of Rheumatology (ACR) [11]. FM management guidelines published by the American Pain Society (APS) [12] and the European League Against Rheumatism (EULAR) [13] reflect that generalized pain does not adequately characterize FM and a broader assessment of pain, function, and psychosocial aspects may aid in FM management. Recognizing the need for a core set of domains that more fully describe FM, the Outcome Measures in Rheumatology Clinical Trials (OMERACT) workgroup on FM was established to identify domains that should be captured in clinical trials for FM therapies [14]. These domains include pain, patient global health, fatigue, health-related quality of life (HRQoL), multidimensional function, sleep, depression, physical function, tenderness, dyscognition, and anxiety.

Due in-part to the chronic nature of the disease, FM patients are high consumers of healthcare services in Europe [15-18]. The lack of a definitive diagnostic test means that FM patients repeatedly present to physicians prior to receiving a confirmed diagnosis. Furthermore, once diagnosed patients often experience suboptimal pain and symptom management which may lead to frequent physician office visits [16]. A review of the literature shows that there are currently no studies that have included a comprehensive assessment of the burden of illness associated with FM in France. The objective of this study was to better understand the impact of FM on French patients by conducting an evaluation of their clinical and comorbid profile, and FM's impact on HRQoL, symptom domains (such as pain, sleep, depression), productivity, and cost to society.

## Methods

### Study design

#### Sample population

This cross-sectional, observational study included FM patients recruited from 18 community-based physician offices (15 general practitioners and 3 rheumatologists) in France. Since there are no definitive criteria for FM diagnosis, patients were required to have a prior diagnosis of FM by a rheumatologist or pain specialist, to have experienced widespread pain (above and below the waist and on both sides of the body) for at least 3 months, and to have experienced pain in the past 24 hours. Patients also were required to have been in treatment at the enrolling physician's practice for at least 3 months. All patients were older than 18 years of age and were excluded if they had participated in an investigational drug study within 30 days prior to the survey or had a concomitant illness unrelated to FM that was likely to confound the assessment of FM. The protocol was approved by central and local accredited ethics committees. No medical interventions or invasive procedures were required by the study protocol. All patients provided written informed consent.

#### Data collection procedures

Site study staff identified potential patients when they presented for routine visits. Once patients provided written informed consent, they were asked to complete a self-administered patient questionnaire. Site staff completed a case report form (CRF) based on review of the patient's medical records and conducted an assessment of FM using the Manual Tender Point Survey (MTPS) exam [19]. Recorded data included patient's clinical characteristics; patient specific pain associated with FM; current and previous medications for FM; concomitant medications prescribed for depression, anxiety, or insomnia; and FM-related office visits, diagnostic tests, and hospitalizations. Information collected via the patient questionnaire and CRF was not associated with a patient's personal identification information but was associated with a study-specific identifier assigned at enrollment to allow linking of individual patients' clinical and survey data in the analysis.

#### Patient questionnaire

The patient questionnaire included five validated instruments that assess the impact of FM on aspects of HRQoL and symptoms (such as pain, sleep, anxiety, and depression): the Fibromyalgia Impact Questionnaire (FIQ) [20], the EuroQol (EQ-5D) [21], the Medical Outcomes Study (MOS) Sleep Scale [22], the Brief Pain Inventory-Short Form (BPI-sf) [23], and the Hospital Anxiety and Depression Scale (HADS) [24]. Patients were asked to rate their current overall health on a scale from 0 to 100, where 0 represents 'worst possible health' and '100' represents 'perfect health'. Patients were also asked to estimate what their overall health status would be, on the same scale, if they had complete relief from FM (pain-free overall health). In addition, study-specific questions were developed to assess perceived treatment effectiveness, treatment satisfaction, and FM impact on productivity and health resource use (HRU).

### Patient perception of treatment effectiveness and satisfaction

To assess perception of FM treatment effectiveness, patients were asked questions relating to prescription medications, non-prescription medications (e.g. over-the-counter medications), and other treatments, including physical treatments (physical therapy/massage, acupressure/acupuncture, chiropracty), and herbs, vitamins, or other supplements. Patients were specifically asked how effective their prescription medications were for relieving their FM symptoms over the past 4 weeks. Response options for any question on effectiveness were: extremely effective, very effective, somewhat effective, a little effective, and not at all effective. Similarly, perceived satisfaction with treatment was assessed through questions relating to prescription medications, non-prescription medications, and other treatments (listed above). Patients were specifically asked how satisfied or dissatisfied they were with the pain relief experienced with their prescription medications over the past 4 weeks. Patients could respond to any question on satisfaction with: extremely satisfied, somewhat satisfied, neither satisfied nor dissatisfied, somewhat dissatisfied, and extremely dissatisfied.

### Health-related quality of life and core symptom domains

The EQ-5D utility score (derived from the health state valuation score) assesses HRQoL across 5 domains, each scored separately: mobility, self-care, performance of usual activities, pain or discomfort, and anxiety or depression [21]. A scoring formula developed by the EuroQol Group is used to assign utility values for each patient's health valuation. Health state valuation scores range from -0.594 to 1.00, where higher scores indicate better health state [21].

The FIQ is a brief 10-item assessment measuring FM patient status, progress, and outcomes in the area of physical impairment, feeling good, work missed, doing work, pain, fatigue/tired, rested, stiffness, anxiety, and depression [20]. Each of the 10 subscales included in the FIQ is scored from 0 to 10. The FIQ total score (range: 0-100) is the sum of the 10 FIQ subscale scores, with higher scores indicating a greater impact of FM on the patient. We utilized patients' FIQ total scores to define FM severity as follows: total scores of 0- < 39 considered mild; total scores of 39- < 59 considered moderate; and total scores of 59-100 considered severe [25].

The BPI-sf includes an evaluation of pain intensity and an evaluation of the interference of pain over the past 24 hours on general activity, mood, walking, work, relationships with others, sleep, and enjoyment of life [23]. The intensity of pain is assessed with four items (pain at its worst, at its least, on average over the past 24 hours, and currently) on an 11-point numeric rating scale ranging from 0 to 10, where higher scores indicate higher severity of pain. The BPI Pain Severity Index score (range: 0-10) is the mean of worst, least, average, and current pain, with previously established cut points for chronic neuropathic pain of 0-3, 4-6, and 7-10 considered mild, moderate, and severe, respectively [26].

The MOS Sleep Scale includes 12 items that measure seven key constructs of sleep: sleep disturbance, snoring, awakening short of breath or with a headache, sleep adequacy, somnolence, optimal sleep, and sleep quantity [22]. With the exception of optimal sleep (1 item; scored as 0 [not optimal] or 1 [optimal]), sleep quantity (1 item; scored as 0-24 per hours of sleep where higher numbers reflect more sleep), and sleep adequacy (2 items; combined score of 0-100, where higher scores indicate greater sleep adequacy), each of the other subscales and the additional 9-item Sleep Problems Index, were scored from 0 to 100 where higher scores represent worse sleep outcomes.

The HADS is designed to assess the presence and severity of mood disorders and has been used extensively in a variety of patient populations [24]. The HADS includes 14 items, of which 7 assess anxiety (HADS-A) and 7 assess depression (HADS-D); subscale scores range from 0 to 21, with higher scores representing more symptoms and poorer emotional well-being. Scores of 0-7 on either subscale are considered normal, 8-10 considered mild, 11-14 considered moderate, and 15-21 considered severe levels of anxiety and depression, respectively.

### Healthcare costs

Annual direct and indirect costs associated with FM were calculated in 2008 Euros using a societal perspective. Costs included direct medical costs (diagnostic tests, physician office visits, prescription medications, hospitalizations, and patient out-of-pocket costs, e.g. from prescription medications, non-prescription medications, and other treatments for FM), direct non-medical-related costs (assistance with activities of daily living), and indirect costs (days missed from work or on disability due to FM).

Costing algorithms were developed to assign 2008 unit costs to each unique type of resource utilized. Unit costs assigned to office visits and office-based procedures were based on current physician fee schedules (i.e. *Classification Commune des Actes Médicaux *[CCAM]) [27]. Unit costs assigned to hospitalizations were based on current hospital case-rate payments (i.e. *Programme de Médicalisation des Systèmes d'Information *[PMSI]) [28]. Medication costs were based on private quotes for current drug price lists (i.e. Thériaque) [29]. Unit costs assigned to days missed from work and disability were based on Eurostat wage data. The average cost of FM, per patient, was summed for the 3-month time horizon based on the data collected, and the mean annual cost of FM, per patient, was calculated based on the 3-month data.

### Statistical analyses

Summary statistics were calculated including mean, standard deviation (SD), median, and range for continuous variables and frequency distributions for categorical variables. Data are given as mean (± SD) unless otherwise indicated. To evaluate the impact of FM severity on patient- and physician-reported outcomes, mean outcomes and costs were compared across FM severity levels (mild, moderate, and severe based on FIQ total scores [25]) the Kruskal-Wallis test. Frequency outcomes were compared across FM severity levels using Fisher's exact test. Statistical significance was evaluated at the 0.05 level, with no adjustments for multiple comparisons. The data were held and analyzed by Covance Inc (Gaithersburg, MD, USA). All analyses were performed using SAS version 9.1 (SAS Institute, Cary, NC, USA).

## Results

### Study sample

Eighty-eight patients from 18 community-based physician offices across France were enrolled. Patients were 55.2 (± 11.8) years and predominantly female (Table 1).

**Table 1:** Demographic characteristics of study sample

		FM Severity			
		
Characteristic	Total (n = 88)	Mild (n = 17)	Moderate (n = 33)	Severe (n = 38)	*P*-value^a^
Age, years					0.303

Mean (SD)	55.2 (11.8)	51.3 (11.3)	56.1 (11.3)	56.2 (12.4)	

Median (range)	57.0 (19.0-80.0)	55.0 (34-69)	57.0 (33-80)	57.5 (19-78)	

Gender, n (%)					1.0

Male	14 (15.9)	3 (17.6)	5 (15.2)	6 (15.8)	

Female	74 (84.1)	14 (82.4)	28 (84.8)	32 (84.2)	

Employment status, n (%)^b^					0.682

Employed, full-time	23 (26.1)	5 (31.3)	11 (33.3)	7 (21.9)	

Employed, part-time	6 (6.8)	1 (6.3)	4 (12.1)	1 (3.1)	

Disabled	8 (9.1)	1 (6.3)	3 (9.1)	4 (12.5)	

Full-time homemaker	2 (2.3)	1 (6.3)	0 (0)	1 (3.1)	

Unemployed	6 (6.8)	2 (12.5)	1 (3.0)	3 (9.4)	

Retired	33 (37.5)	5 (31.3)	13 (39.4)	15 (46.9)	

Other	2 (2.3)	1 (6.3)	1 (3.0)	0 (0)	

Student	1 (1.1)	0 (0)	0 (0)	1 (3.1)	

Patient Survey

^a^Fisher's exact test or Kruskal-Wallis test, as appropriate

^b^Percentages for each column might not add up to 100% due to missing data on given question.

Patients had been diagnosed with FM for 3.2 (± 2.9) years, on average, and half (52.3%) reported FM symptoms for 1-5 years (Table 2). The average number of MTPS points was 13.0 (± 3.4). Patients had an average of 3.4 comorbid conditions, with common (reported by > 25% of patients) comorbidities being anxiety (76.1%), sleep disturbance/insomnia (59.1%), and chronic fatigue syndrome (52.3%).

**Table 2:** Clinical characteristics of overall sample

		FM Severity^a^			
		
Characteristic	Total (n = 88)	Mild (n = 17)	Moderate (n = 33)	Severe (n = 38)	*P*-value^b^
Duration of FM symptoms^c^, n (%)					0.517

3-6 months	10 (11.4)	3 (17.6)	2 (6.1)	5 (13.2)	

7-11 months	4 (4.5)	0 (0.0)	2 (6.1)	2 (5.3)	

1-5 years	46 (52.3)	8 (47.1)	22 (66.7)	16 (42.1)	

6-10 years	20 (22.7)	5 (29.4)	4 (12.1)	11 (28.9)	

> 10 years	7 (8.0)	1 (5.9)	3 (9.1)	3 (7.9)	

Time since diagnosis, years					0.983

Mean (SD)	3.2 (2.9)	3.2 (2.5)	3.1 (2.6)	3.4 (3.3)	

Median	2	3	2	2	

Number of positive MTPS points					0.165

Mean (SD)	13.0 (3.4)	14.4 (2.9)	12.6 (3.1)	12.7 (3.9)	

Median	13	15	12	12	

Comorbid conditions^d^					

Anxiety, n (%)	67 (76.1)	12 (70.6)	26 (78.8)	29 (76.3)	0.808

Sleep Disturbance/Insomnia, n (%)	52 (59.1)	10 (58.8)	22 (66.7)	20 (52.6)	0.477

Chronic Fatigue Syndrome, n (%)	46 (52.3)	13 (76.5)	17 (51.5)	16 (42.1)	0.055

Depression, n (%)	40 (45.5)	6 (35.3)	15 (45.5)	19 (50.0)	0.618

Headache/Migraine, n (%)	30 (34.1)	6 (35.3)	7 (21.2)	17 (44.7)	0.113

Restless Leg Syndrome, n (%)	22 (25.0)	8 (47.1)	8 (24.2)	6 (15.8)	0.043

Irritable Bowel Syndrome, n (%)	16 (18.2)	3 (17.6)	4 (12.1)	9 (23.7)	0.450

Raynaud's Syndrome, n (%)	4 (4.5)	1 (5.9)	1 (3.0)	2 (5.3)	1.00

Other, n (%)	6 (6.8)	1 (5.9)	1 (3.0)	4 (10.5)	0.562

Number of comorbid conditions, n (%)					0.934

0	5 (5.7)	1 (5.9)	1 (3.0)	3 (7.9)	

1	15 (17.0)	2 (11.8)	5 (15.2)	8 (21.1)	

2	16 (18.2)	4 (23.5)	6 (18.2)	6 (15.8)	

≥3	52 (59.1)	10 (58.8)	21 (63.6)	21 (55.3)	

Number of comorbid conditions^e^					0.637

Mean (SD)	3.4 (1.8)	3.8 (2.0)	3.2 (1.5)	3.5 (2.1)	

Median (range)	3 (1-8)	4 (1-7)	3 (1-6)	3 (1-8)	

Clinical case report form.

^a ^Some data were not available, and therefore N-numbers for each group represents the maximum number of patients.

^b ^Fisher's exact test or Kruskal-Wallis test, as appropriate.

^c ^Percentages for each column might not add up to 100% due to missing data.

^d ^Categories are not mutually exclusive.

^e ^Among patients reporting at least 1 comorbid condition

### Patients' perception of treatment effectiveness and satisfaction

All patients were actively seeking care for their FM. Patients made 2.9 (± 1.9) office visits to the study site over the past 3 months (Table 3). One-quarter (25.0%) of patients also made visits to other physicians' offices. The more common physician specialties visited by patients for their FM were rheumatologist (54.5%), general practitioner (GP) (18.2%), neurologist (13.6%), and surgeon (13.6%).

**Table 3:** Number of physician visits for FM cohort, and stratified by FIQ-based FM severity

		FM Severity			
		
Characteristic	Total (n = 88)	Mild (n = 17)	Moderate (n = 33)	Severe (n = 38)	*P*-value^a^
Number of office visits over the past 3 months					0.319

Mean (SD)	2.9 (1.9)	3.1 (1.3)	2.5 (1.3)	3.3 (2.4)	

Median	3	3	3	3	

Other physician visits over the past 3 months^b^, n (%)					0.379

Yes	22 (25.0)	2 (12.5)	10 (31.3)	10 (27.0)	

No	63 (71.6)	14 (87.5)	22 (68.8)	27 (73.0)	

Clinical case report form.

^a ^Fisher's exact or Kruskal-Wallis test, as appropriate.

^b ^Percentages for each column might not add up to 100% due to missing data.

**Figure 1:**
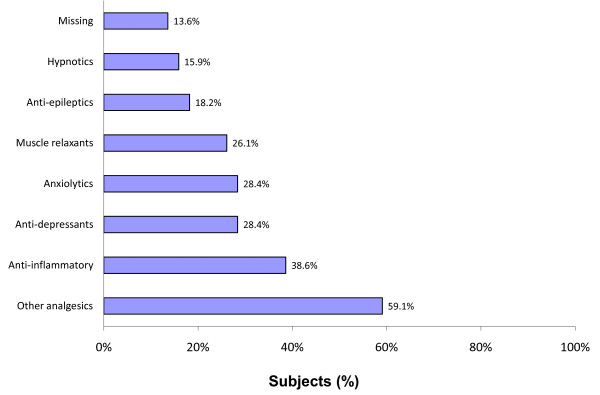
**Percent of patients on medication, by class, over the past 3 months Clinical case report form**. Patients may be prescribed multiple medications so the categories are not mutually exclusive. The percent of patients receiving prescription medications for FM was 84.1%.

**Figure 2:**
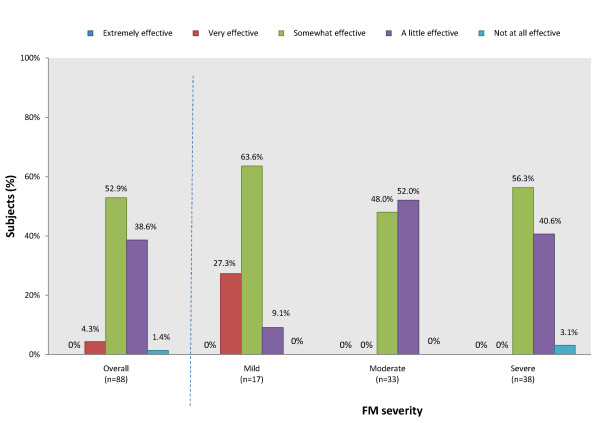
**Patient-reported perceived effectiveness of prescription medications for their FM**. Patient Survey. Numbers may not add up to 100%. Fisher's exact test performed across severity levels (*P *= 0.008)

**Figure 3:**
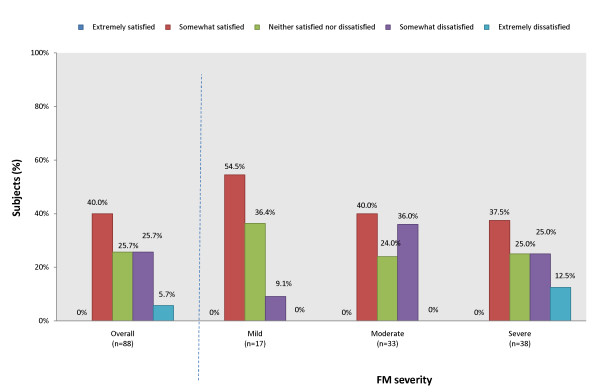
**Patient-reported satisfaction with prescription medications for their FM**. Patient Survey, numbers may not add to 100%. Fisher's exact test performed across severity levels (*P *= 0.350)

Most patients had received prescription medications for their FM within the past 3 months (n = 74/88). Among those receiving prescription medications for FM, one or a combination of the following classes of medications were prescribed: analgesics (59.1%), anti-inflammatories (38.6%), antidepressants (28.4%), anxiolytics (28.4%), and muscle relaxants (26.1%) (Figure 1). Assessment of patient's perceived effectiveness of their FM prescription medications found that no patient reported that their prescription medications were 'extremely effective' (Figure 2). For patients who responded as taking a prescription medication for FM within the past 4 weeks (n = 70), the majority (52.9%) reported medications as being 'somewhat effective' and 38.6% reported their prescription medications as being 'a little effective' (Figure 2). Assessment of patient's satisfaction with their FM prescription medications found that no patient reported being extremely satisfied (Figure 3).

### Impact of fm on health-related quality of life and core symptom domains

With respect to overall functioning and well-being, patients had an average EQ-5D health state score of 0.37 (± 0.33) (Table 4). The Current Overall Health rating was 52.9 (± 17.8), and patients estimated their Pain-Free Overall Health 27.7 points higher (80.6) if they had complete relief from FM-related pain (Table 4).

**Table 4:** HRQoL assessment scores, for whole cohort and stratified by FIQ-based FM severity

Scale	Total (n = 88)	Mild (n = 17)	Moderate (n = 33)	Severe (n = 38)	*P*-value^a^
EQ-5D					

Health State valuation	0.37 (0.33)	0.65 (0.18)	0.44 (0.27)	0.18 (0.33)	< 0.001

Overall Health Status Ratings					

Current Overall Health	52.9 (17.8)	65.4 (14.2)	53.0 (11.9)	47.7 (20.9)	0.006

Pain-Free Overall Health	80.6 (19.2)	86.9 (12.8)	80.5 (22.6)	77.9 (17.6)	0.106

FIQ					

Total Score	54.8 (17.3)	29.3 (8.8)	50.0 (5.5)	70.4 (8.5)	

Physical Impairment	3.4 (2.1)	1.6 (1.9)	3.2 (2.0)	4.5 (1.7)	

Feel Good	7.1 (2.8)	4.4 (2.9)	7.6 (2.5)	8.0 (2.3)	

Work Missed	3.0 (3.5)	0.6 (1.9)	1.1 (2.1)	5.6 (3.3)	

Do Work	6.0 (2.3)	3.2 (1.3)	5.4 (1.4)	7.6 (1.9)	

Pain	6.2 (2.2)	3.2 (1.4)	5.9 (1.4)	7.9 (1.5)	

Fatigue/Tired	6.9 (2.1)	4.7 (1.9)	6.7 (1.6)	8.3 (1.3)	

Rested	6.8 (2.5)	4.3 (2.3)	6.5 (2.2)	8.1 (1.7)	

Stiffness	6.6 (2.4)	4.0 (2.0)	6.5 (2.1)	8.0 (1.8)	

Anxiety	5.3 (2.7)	2.4 (1.9)	4.7 (2.0)	7.1 (2.0)	

Depression	4.3 (3.2)	1.2 (1.4)	3.2 (2.4)	6.6 (2.7)	

BPI-sf					

Pain Intensity: Worst	6.2 (2.0)	4.4 (1.9)	6.0 (1.4)	7.3 (1.8)	< 0.001

Average pain	4.8 (1.9)	3.1 (1.7)	4.6 (1.3)	5.7 (1.8)	< 0.001

Pain Severity Index	4.9 (1.8)	3.2 (1.4)	4.7 (1.2)	6.0 (1.7)	< 0.001

Pain Interference Index	5.1 (1.9)	3.0 (1.1)	4.6 (1.2)	6.6 (1.6)	< 0.001

General activity	5.6 (2.1)	3.7 (1.7)	5.3 (1.4)	6.8 (2.1)	< 0.001

Mood	4.8 (2.6)	2.9 (2.3)	4.2 (2.1)	6.3 (2.3)	< 0.001

Walking ability	4.6 (2.7)	2.2 (1.5)	4.2 (2.3)	6.0 (2.6)	< 0.001

Normal work	6.1 (2.0)	3.7 (1.1)	5.6 (1.4)	7.6 (1.5)	< 0.001

Relations with other people	4.0 (2.6)	1.9 (1.8)	3.2 (2.3)	5.7 (2.2)	< 0.001

Sleep	5.5 (2.9)	3.8 (2.8)	4.5 (2.5)	7.1 (2.4)	< 0.001

Enjoyment of life	5.1 (2.4)	2.9 (1.7)	4.7 (2.2)	6.5 (2.0)	< 0.001

MOS					

Sleep Problems Index	52.8 (16.8)	45.9 (20.5)	48.6 (14.9)	59.5 (14.4)	0.008

Sleep disturbance	53.8 (25.1)	47.6 (27.8)	49.8 (24.6)	60.1 (23.6)	0.106

Sleep adequacy	34.2 (24.1)	42.4 (24.4)	34.6 (22.9)	30.3 (24.7)	0.201

Sleep somnolence	43.3 (22.6)	38.8 (26.7)	36.0 (18.9)	51.6 (21.4)	0.006

Snoring	43.9 (31.2)	38.7 (29.7)	36.4 (28.0)	53.5 (32.9)	0.074

Sleep SOB or headache	35.2 (26.9)	25.0 (33.9)	27.3 (23.4)	46.3 (22.8)	0.001

Sleep quantity (hours)	6.4 (1.7)	6.5 (1.6)	6.8 (1.4)	6.0 (1.9)	0.092

HADS					

Anxiety	10.4 (4.1)	8.0 (4.1)	9.9 (2.9)	11.9 (4.4)	0.002

Depression	7.9 (4.3)	4.7 (3.0)	6.6 (3.3)	10.5 (4.0)	< 0.001

Patient Survey

^a^Kruskal-Wallis test.

SOB, shortness of breath

FIQ total score was 54.8 (± 17.3) (Table 4), with 17 (19.3%), 33 (37.5%), and 38 (43.2%) patients reporting mild (scores 0- < 39), moderate (scores 39- < 59), and severe scores (scores 59-100), respectively. FIQ domains most affected (> 6.0) were feel good (7.1), fatigue/tired (6.9), rested (6.8), stiffness (6.6), and pain (6.2) (Table 4).

BPI-sf Pain Severity Index was 4.9 (± 1.8) (Table 4), with 54.5% of the patients reporting moderate pain (scores 4-6) and 21.6% reporting severe pain (scores 7-10). Pain Interference Index was 5.1 (± 1.9) (Table 4). BPI domains most affected (score > 5) were normal work (6.1), general activity (5.6), sleep (5.5), and enjoyment of life (5.1) (Table 4).

Assessment of the sleep using the MOS found average MOS Sleep Problems Index score of 52.8 (± 16.8), and sleep quantity of 6.4 (± 1.7) hours (Table 4). The MOS domain most affected was sleep adequacy (34.2 [± 24.1]), where higher scores indicate greater sleep adequacy.

Assessment of anxiety using the HADS-A found patients had average score of 10.4 (± 4.1) (Table 4). More specifically, 25.0% of patients showed normal anxiety (scores 0-7), 25.0% mild anxiety (scores 8-10), and 35.2% moderate (scores 11-14) levels of anxiety. Assessment of depression using the HADS-D found average score of 7.9 (± 4.3) (Table 4), with 52.3%, 19.3%, and 21.6% of the patients reporting normal (scores 0-7), mild (scores 8-10), and moderate (scores 11-14) levels of depression, respectively.

### Impact of fm on productivity

Some level of disruption to their employment status due to their FM was reported by almost half (44.3%) of all patients, including having to reduce their work time (18.2%), becoming disabled (13.6%), or becoming unemployed or taking early retirement (12.5%). Among those employed full- or part-time, all reported some degree of reduced productivity while at work with an average of 2.7 days of work missed in the previous 4 weeks (Table 5).

**Table 5:** Impact of FM on productivity among patients employed for pay

		FM Severity			
		
Characteristic	Total (n = 29)	Mild (n = 6)	Moderate (n = 15)	Severe (n = 8)	*P*-value^a^
Days missed from work during the past 4 weeks^b^					0.309

n	27	6	13	8	

Mean (SD)	2.7 (6.0)	0.5 (1.2)	1.2 (2.8)	6.9 (9.6)	

Median (range)	0 (0-20)	0 (0-3)	0 (0-8)	0 (0-20)	

Reduced productivity at work during the past 4 weeks^b^, n (%)					0.281

All of the time	4 (13.8)	0 (0.0)	2 (13.3)	2 (25.0)	

Most of the time	4 (13.8)	0 (0.0)	3 (20.0)	1 (12.5)	

A good bit of the time	9 (31.0)	1 (16.7)	4 (26.7)	4 (50.0)	

Some of the time	11 (37.9)	4 (66.7)	6 (40.0)	1 (12.5)	

A little of the time	1 (3.4)	1 (16.7)	0 (0.0)	0 (0.0)	

None of the time	0 (0.0)	0 (0.0)	0 (0.0)	0 (0.0)	

Patient Survey.

^a^Fisher's exact test or Kruskal-Wallis test, as appropriate.

^b^Among patients who are employed (full-time or part-time).

### Impact of fm on healthcare costs

Total annual costs per patient for treating FM in France was €7,900 (€14,868), comprising direct costs of €910, and indirect costs of €6,990 (Table 6). Lost productivity accounted for the majority of costs (~88.5% total costs [direct+indirect]) associated with FM. The major cost drivers for the direct medical costs were payer costs for physician office visits (€259/€808 [32.1%]) and payer costs for prescription medications (€245/€808 [30.3%]). Patient direct medical costs (€186) accounted for 23.0% of direct medical costs (Table 6).

**Table 6:** Annual costs (2008 Euros) per FM patient, and by FM severity

Resource Utilization Cost	Total (n = 88)		Mild (n = 17)		Moderate (n = 33)		Severe (n = 38)		
		
	Mean (SD)	Median	Mean (SD)	Median	Mean (SD)	Median	Mean (SD)	Median	***P*-value**^a^
Direct Medical Costs to Payer^b^									

Physician Visits	259 (163)	264	271 (118)	264	219 (112)	264	288 (208)	264	

Diagnostic Tests	14 (38)	0	26 (61)	0	7 (20)	0	13 (37)	0	

Prescription Medications	245 (345)	107	153 (207)	43	213 (312)	102	314 (409)	144	

Hospitalizations	104 (972)	0	0 (0)	0	276 (1,587)	0	0 (0)	0	

Direct Medical Costs to Patient^c^	186 (425)	0	113 (256)	0	233 (448)	0	179 (467)	0	

Total Direct Medical Costs to Society	808 (1,215)	540	564 (440)	528	949 (1,842)	504	794 (636)	595	0.434

Direct Non-Medical Cost to Patient^c^									

Professional services for ADL	103 (501)	0	93 (274)	0	108 (428)	0	103 (633)	0	

Total Indirect Costs to Society^c^									

Lost productivity	6,990 (14,561)	0	4,816 (12,433)	0	5,576 (12,736)	0	9,190 (16,808)	0	

Total Direct and Indirect Costs to Society	7,900 (14,868)	616	5,473 (12,852)	610	6,633 (13,498)	528	10,087 (16,785)	890	0.185

Extrapolated from 4-week patient-reported data and 3-month clinical case report form data

^a^Kruskal-Wallis test.

^b^Based on unit cost and data reported in Clinical case report form.

^c^Based on patient-reported data from study.

ADL: activities of daily living

### Impact of FM severity

Baseline characteristics were similar across FM severity cohorts, including age, gender, and employment status (Table 1). Almost half (15/32 [46.9%]) of the severe FM patients were retired and a further 21.9% were disabled or unemployed. Time since diagnosis of FM was similar across patients in different severity cohorts (~3 years) (Table 2). None of the clinical characteristics differed significantly across FM severity levels (Table 2) except for the proportion of patients with restless leg syndrome (RLS), which was highest in patients with mild FM (47.1%) (*P *= 0.043). Interestingly, more than 70% of patients in each FM severity cohort reported anxiety, and at least 50% in each severity cohort reported sleep disturbance/insomnia.

With regard to healthcare visits, there was no association between FM severity and number of office visits within the past 3 months (Table 3) An association was found between FM severity level and the EQ-5D health state valuation score (mild: 0.65, moderate: 0.44, severe: 0.18; *P *< 0.001), where poorer overall health status was associated with more severity (Table 4) Additionally, there was an association between FM severity level and current overall health scores, where current overall health score decreased from 65.4 to 53.0 to 47.7 for mild to moderate to severe FM, respectively (*P *= 0.006) (Table 4). As might be expected, an association was also found between FM severity level and BPI-sf Pain Severity Index scores from 3.2 for patients with mild FM, 4.7 with moderate FM, and 6.0 with severe FM (*P *< 0.001)(Table 4). Pain interference index also differed across FM severity level, from 3.0 for patients with mild FM to 4.6 for those with moderate FM, and to 6.6 for patients with severe FM (*P *< 0.001).

An association between FM severity level and certain sleep outcomes was found (Table 4). For example, MOS Sleep Problems Index increased as FM FIQ severity increased, from 45.9 for patients with mild FM, to 48.6 for patients with moderate FM, and 59.5 for severe FM (*P *= 0.008). Using the HADS-A patients reported increasing anxiety as FM severity worsened from mild (8.0) to moderate (9.9) to severe (11.9) (*P *= 0.002). The HADS-D scores showed a similar trend indicating increasing depression as the FM severity level worsened (4.7, 6.6, 10.5, respectively; *P *< 0.001) (Table 4). Comparisons across FM severity level for the FIQ are not made because FM severity level was developed from the FIQ total score.

Nearly three-quarters (74.2%) of patients in the severe FM cohort reported FM-related disruptions in employment status. Although not significant, the average number of days missed from work due to FM during the past 4 weeks was 0.5 days for mild, 1.2 days for moderate, and 6.9 days for severe FM (*P *= 0.309) (Table 5). There also was no significant relationship between FM severity level and at-work productivity over the past 4 weeks.

As FM severity level worsened, patients perceived their prescription medications to be less effective (Figure 2) (*P *= 0.008 for association across cohorts). Patients also generally became less satisfied with their prescription medication as severity level worsened (Figure 3), although the association was non-significant.

Average annual total cost (direct and indirect) per FM patient was higher for patients with severe FM (€10,087) compared to those with moderate (€6,633) or mild FM (€5,473); however, the difference was not significant (Table 6). As observed overall, lost at-work productivity was the main driver of total costs within each severity level.

## Discussion

This is the first study to assess core FM symptom dimensions identified by OMERACT, in a group of 88 patients with FM from France. We examined FM burden of illness by investigating patients' tender points, HRQoL, general health, pain, sleep/fatigue, depression, anxiety, physical function, productivity losses for FM patients, medication use, treatment satisfaction, as well as the costs to society. Consistent with other studies [4,30-32], our study demonstrated that patients have substantial burden due to FM, and FM is associated with direct and indirect costs.

The results of this study show a significant burden of illness associated with FM. FM severity was associated with certain sleep problems, anxiety, and depression. Patients reported poor HRQoL, overall and in negative health impact in relation to measures of pain, function, sleep, anxiety, and depression. Just over half (55%) of patients had moderate pain based on the BPI-sf Pain Severity Index, and 22% severe pain, supporting that moderate-to-severe chronic pain is a dominant feature for patients with FM.

Although the generalizability of our observations from these FM patients to the wider FM population in France is ultimately unknown, our results are consistent with other larger studies that have examined the impact of FM. For example, a Dutch study involving a sample of 3664 patients and examining the impact of musculoskeletal diseases on HRQoL, reported that patients with FM (with or without other musculoskeletal diseases) scored lower on all subscales of the Short Form-36 (SF-36) and EQ-5D health status measures than study patients with other musculoskeletal diseases [33]. The domains most affected were vitality, role-physical, and bodily pain for the SF-36; and usual activities and pain/discomfort for the EQ-5D. In a study of chronic widespread pain among patients with and without FM in Sweden, the FM group scored significantly lower than the non-FM group on general HRQoL measures and specific measures for activities of daily living, depression, anxiety, and pain [34]. Collectively with our study, data highlight the high societal and patient burden that FM inflicts across Europe [35].

Patients in our study reported a significant impact of FM on sleep, with the most affected areas on the MOS Sleep Scale being sleep adequacy and sleep disturbance. These observations are consistent with other larger studies of European patients with FM, and FM patients from other countries [3,36] For example, in a study of 600 health maintenance organization (HMO) members with FM [3], patients demonstrated poor sleep quality as measured by the Pittsburgh Sleep Quality Index (PSQI) where scores of ≥5 indicates poor sleep. More specifically, Bigatti et al. reported patients had PSQI score of 11.22 (± 3.96) at baseline, with only 4% of patients scoring < 5 [3].

FM had a negative impact on work productivity in the present study, with 44% of patients reporting some disruption in productivity, and the overall employed sample reporting an average of 35 days of missed work per year per patient. Thirty-five days of missed work due to FM accounts for approximately 13% of all working days in a calendar year. Other studies have likewise highlighted the higher number of work days missed for employed patients with FM vs. the general population, in Europe and the United States. For example, in a similar small study of patients with FM, chronic low back pain, and ankylosing spondylitis, Boonen et al. (2005) found that 63% of FM patients with a paid job reported an episode of sick leave, with mean length of sick leave was 34 days per working-patient-year [15]. In a larger study of administrative claims database including 31 large self-insured companies in the United States, White et al. (2005) reported that FM patients missed significantly more days of work in the past year compared with non-FM patients (29.8 vs. 10.4 days; *P *< 0.001) [4]. Similarly, a study of 1081 patients with FM from Spain reported 20.9 sick days per year, significantly more than the reference group of subjects without FM syndrome (8 days) [32].

Patients reported an average of one physician office visit per month in the present study. Physicians reported that 84% of patients were taking a prescription medication related to their FM symptoms, with the majority of patients taking other analgesics, primarily opioids, and non-steroidal anti-inflammatory drugs. Furthermore, patients did not perceive their prescription medications as completely effective and expressed some dissatisfaction with current pharmacological treatments. Our findings suggest that there is room for improvement in the current management and use of prescription medications for FM in France. These observations are relatively consistent with other larger studies of European populations of FM patients. For example, a study of 299 patients with FM from France and Germany reported that their FM treatment regimens were not the most advantageous [18]. Other European studies have also documented frequent physician office services among patients with FM. In a UK study using a large electronic medical records database containing data on GP visits, Hughes et al. (2006) reported that, among 2,260 UK patients newly diagnosed with FM, there were 25 office visits, and 11 prescriptions per patient in the year prior to diagnosis, and levels of HRU generally increased following diagnosis [16]. Using a large electronic database recording GP encounters in Germany, Berger et al. (2008) also demonstrated significant HRU for FM patients. Among 4,983 FM patients, 67% were on at least one pain-related medication and 74% had four or more GP office visits over 1 year. Additionally, FM patients averaged approximately three-times as many outpatient office visits (19.6 vs. 5.2; *P *< 0.001) than patients without FM [17]. Similarly, a claims analysis from Spain documented an average of 13.5 GP office visits per year and the use of an annual average of 3.7 medications for 1081 FM patients [32]. Taken with the data presented in the current study, despite diagnosis and treatment, FM patients display considerable HRU across Europe, indicating an unmet need for FM patients in these studies.

Higher HRU rates resulted in higher total direct medical costs on a per-patient basis. The major drivers of direct medical costs to the payer were physician office visits and prescription medications. The largest contribution to FM costs in our study was related to lost productivity due to absenteeism and disability, accounting for approximately 88% of total costs. These results are supported by the published literature. Previous studies have demonstrated that employee disability and medical comorbidity associated with FM greatly increase the economic burden of the disease. White et al. (2008) reported that indirect costs, including actual employer payments for extended absence from work due to disability and imputed medically-related work-loss days and costs, accounted for approximately one-third of the total study costs, highlighting the significant burden imposed by FM to employers [4]. Robinson et al. (2003) also found that a substantial portion of total cost for FM was due to work disability; the prevalence of disability was twice as high among employees with FM when compared with the overall employee population [37].

The present study is the first to assess the impact of FM severity on key multiple FM dimensions. Eighty-one percent of the sample reported moderate (38%) or severe FM (43%) based on patients' FIQ total scores and the results of the study also show that, as FM severity increased, facets of patients' HRQoL, pain, elements of sleep, anxiety, and depression worsened significantly. In addition, there was a non-significant trend for patients' productivity and the total direct and indirect costs to society to increase as FM severity worsened.

This study had several limitations. Firstly, only practices that volunteered to participate were included in the study. It is possible that these sites may differ in unknown ways from others that routinely provide FM or general patient care. Additionally, these practices were predominantly GPs, and it is possible that FM patients presenting to GPs are not generalizable to the FM patient population in France as a whole. However, we note that GPs are the most accessible venue for FM patients to seek care, and a large proportion of patients were concurrently seeking care for their FM from other specialists. Data came from patients who were actively seeking care; the clinical/sociodemographic characteristics of FM patients who were not seeking care are not known, and our findings may not be generalizable to the wider FM patient population. It is possible that differences were not significant because the low number of patients in each severity cohort is underpowered to detect differences. Therefore these outcomes warrant further investigation in studies with larger sample sizes. Although each scale used in the present study has been independently validated to assess given symptoms in different patient populations, they have not all been validated in FM patients specifically. However, the scales picked are widely used in FM clinical trials to assess subjective outcomes, and some scales are recommended to healthcare providers for assessment of symptoms. Our costing algorithm made several assumptions that may have underestimated medication costs, including costing based on generic medications, determining costs using the largest package sizes available, and assuming lowest average dose where information from the CRF was incomplete. A less conservative costing methodology might have led to higher estimated costs associated with medications. While we captured out-of-pocket costs incurred by patients for non-prescription medications and services by allied healthcare professionals beyond the GP/hospital environment, these types of services and costs often recommended to FM patients, such as physiotherapists, exercise specialists, or psychotherapists, may have been underreported and increased the cost burden to patients or society as a result. Finally, the study was cross-sectional; therefore, while we can examine the association between FM and outcome measures, directionality cannot be established. Despite these limitations, given the lack of information on the burden of FM in patients from France; our study provides important insights into the impact of FM in this sample of patients with FM from France.

## Conclusion

This study represents one of the first attempts to characterize the full patient experience of disease, function, HRQoL, and costs of patients with FM from France. Although the majority of patients were receiving medical attention and prescription medications for FM, patients still reported high levels of pain, anxiety, depression, sleep disturbance, diminished HRQoL, and substantial loss of productivity. Additionally, patients reported that prescription medications for their FM were not optimal in terms of perceived effectiveness and satisfaction. As FM severity increased, patients' health status and other key symptom domains worsened. Finally, the total direct and indirect costs to society increased as FM severity increased. These results highlight the significant disease burden as well as limitations of treatment options available.

## Competing interests

Professor SP has conducted two studies in fibromyalgia for Pfizer Inc as a national coordinator and has received fees for his coordination. CS and TK are employees of Covance Market Access Services Inc., and served as paid consultants to Pfizer Inc in relation to conduct of the study. MH was an employee of Covance Market Access Services, Inc., at the time of the study, and served as a paid consultant to Pfizer in relation to the conduct of the study. AC and GZ are employees of Pfizer Inc.

## Authors' contributions

SP served as clinical reviewer, providing interpretation and critical revision to the analysis and manuscript. CS, ABC, and GZ conceptualized the study design, and together with TK and MH, contributed to the analysis of study results. All authors contributed to the development of the manuscript, and reviewed and approved the final draft.

## Pre-publication history

The pre-publication history for this paper can be accessed here:

http://www.biomedcentral.com/1471-2474/13/22/prepub
